# Incidence and pattern of injuries among adolescent basketball players in Nigeria

**DOI:** 10.1186/1758-2555-4-15

**Published:** 2012-05-04

**Authors:** Oluwatoyosi Babatunde Alex Owoeye, Ashiyat Kehinde Akodu, Bayonle Matt Oladokun, Sunday Rufus Akinwumi Akinbo

**Affiliations:** 1 Department of Physiotherapy, College of Medicine, University of Lagos, Idi-Araba, Lagos, Nigeria

## Abstract

**Background:**

Basketball is the second most popular sport in Nigeria after football and is commonly played by adolescents. Prospective studies on adolescent basketball players in Nigeria are lacking. Such studies will help to develop injury counter-measures. Hence, this study aimed at determining the incidence and pattern of injuries among adolescent basketball players in Nigeria.

**Methods:**

A prospective observational study involving 141 adolescent basketball players (75 boys and 66 girls; with age range 15 – 18 years) who participated in the 2010 National Finals of the Nigeria Nestlé Milo Basketball Competition. Basketball-related injury data were collected by an assessor during the competition using a standardized basketball injury report form. Data were analyzed using descriptive and inferential statistics.

**Results:**

A total of 32 injuries were recorded with an incidence of 22.7 injuries per 100 participants same for boys and girls. This is equivalent to 1.1 injury per match for boys and 0.9 injuries per match for girls. Jumping/landing was the most common cause of injury (28.1%, N = 9). Most of the injuries were at the lower extremities (75%, N = 24); with majority at the knee joint (40.6%, N = 13). Ligament sprain was the most common types of injury. The pattern of injuries among boys did not significantly differ from that of girls (P > 0.05). Most injuries (N = 13, 41%) occurred in the offensive half of the court and cryotherapy was the most frequently used treatment modality.

**Conclusion:**

The overall incidence of match injury among adolescent amateur basketball players during a national competition in Nigeria was 22.7 injuries per 100 participants; equivalence of 1.0 injury per match. The pattern of injuries was similar in both genders and consistent with what has been previously reported in literature for adolescent basketball players. Exercise-based injury prevention programmes aimed at improving core strength and neuromuscular control at the lower limbs may help reduce the incidence of injuries.

## Background

Youth sports injury is a public health concern, as it has detrimental effects on the health and well being of young athletes [1]. Increasingly, competitive youth sports around the world require adolescents to undertake more prolonged and intensive training programmes [2]. Increased playing is mirrored by an increased risk of traumatic injury or overuse musculoskeletal injury by young sports participants [1]. It has been established that specific injury pattern occur in different sports and at different levels [3,4]. Basketball continues to increase in popularity at all levels of play, from recreational to professional and remains immensely popular, not just in the United States, but throughout the world [5].

Basketball appears to be the second most popular sport in Nigeria after football (soccer). Most public and private schools in Nigeria have standard basketball courts where students play during break time and after school. Epidemiological studies on basketball injuries in Nigeria are sparsely documented compared to the United States and Europe. Literature review showed that only one study has investigated the pattern of basketball injuries in Nigeria [6]. The study was limited to professional male basketball players only. It reported an injury incidence of 0.7 injury per match. It further revealed that the ankle followed by the knee were the most frequently injured body parts, while sprain and contusion were the most common types of injuries sustained. Most of the injuries occurred in the offensive zone of the court and collision was the most common causes of injuries [6]. In another study focused on several sports, basketball players among other athletes were reported to have the highest injury rate [7].

Overall incidence rate in basketball have been reported to be higher during competitive matches than training sessions [8,9]. Results of the most commonly studied population of adolescent basketball players – American public high school players, revealed that injury incidence vary considerably, from 5.6 – 36.8 per 100 participants for boys and 7.8 – 49.0 per 100 participants for girls [9].

There appears to be a dearth of study on the incidence and pattern of basketball injuries among adolescent players in Nigeria. The study aimed at determining the incidence and pattern of injuries among adolescent male and female basketball players in Nigeria. The study also investigated the types of first aid treatments rendered to the injured players.

## Methods

### Participants and setting

A cohort of 141 basketball players who were duly registered for the national finals of the Nigeria Nestlé Milo Secondary Schools Basketball Competition participated in the study. A total of 32 matches were played and all the matches were prospectively followed. The competition was held at the indoor sports hall of the National Stadium, Surulere, Lagos State, in October, 2010.

The Nestlé MILO Secondary School Basketball Competition is an annual event sponsored by MILO in partnership with the Nigerian School Sports Federation. The programme is targeted at students aged 13 – 18 years. The 12^th^ edition of the competition had a total of 4,252 schools nationwide who participated from the state preliminary stage to the national finals. These secondary schools across the 6 geopolitical zones/conferences of the country were drawn into groups in the various conferences namely: the Savannah Conference, Central Conference, Atlantic Conference, Western Conference, Sahara Conference and Equatorial Conference. Male and female basketball teams competed in playoffs at the various conferences to produce finalists. Two schools emerged from each of the zones in the boys and girls categories which then made the 12 basketball teams (6 for each gender) that participated in the national finals of the competition.

### Instrumentation

A standardized basketball injury report form [6] specifically designed for basketball was used for the study. It contained information regarding demographic data, cause of the injury, type of injury, body part injured, first aid rendered, the zone of the court where injury occurred and whether the player returned to the court of play after treatment. Detailed injury reports describing all the aforementioned were completed by the attending physiotherapist who was also the assessor for each injury.

### Procedure for data collection

The physiotherapist (a member of the study group) was present at all matches of the competition to attend to injured players and document injuries. An injury was documented when an injured player required at least minimum on-field (medical) care such as ice, tape, etc. regardless of whether the player was able to continue or not (that is both medical attention and time-loss injuries were recorded). All the participating schools gave their consent before the commencement of the competition and ethical approval to conduct the study was also sought for and obtained from the research and ethics committee of the Lagos University Teaching Hospital.

### Data analysis

Descriptive statistics of frequency and percentage were used. Inferential statistics of chi-square was used to determine significant difference between certain variables. Incidence of injuries was calculated per 100 participants thus – total number of injuries divided by the total number of players that participated (separately calculated for boys, girls and overall). Incidence was also calculated per match. For all statistical tests, the level of significance was taken at P ≤ 0.05.

## Result

### Participants

There were 141 players comprising 75 boys (53.2%) and 66 girls (46.8%) aged 16.3 ± 0.9 years (range of 15 – 18 years).

### Incidence

A total of 32 injuries (17 for boys and 15 for girls) were recorded during the competition. The overall incidence of injury was 22.7 injuries per 100 participants for both genders. An incidence of 1.1 injuries per match and 0.9 injuries per match were recorded for boys and girls respectively. The overall incidence was 1.0 injury per match.

### Causes of injuries

“Jumping/landing” was found to be the most common cause of injury, accounting for 9 cases (28.1%) while “being hit by a projectile ball” was the least cause of injury (N = 1, 3.1%) (Table 1).

**Table 1:** Causes of injuries

**Causes of injuries**	**Boys n (%)**	**Girls n (%)**	**Total n (%)**
Collision/contact with obstacle	3 (60.0)	2 (40.0)	5 (15.6)
Jumping/landing	4 (45.0)	5 (55.0)	9 (28.1)
Hit by a projectile ball	1 (100.0)	- (0.0)	1 (3.1)
Previous injury	5 (71.4)	2 (28.6)	7 (21.9)
Sudden turn, twist or stop	4 (50.0)	4 (50.0)	8 (25.0)
Others	- (0.0)	2 (100.0)	2 (6.3)
Total	17	15	32 (100.0)

*X*^2^ – 4.49, p = 0.481.

### Types of injuries and body parts affected

Sprain was found to be the most common type of injury accounting for 62.5% of all injuries (Table 2). There was no statistically significant difference in the mean percentage difference in the number of boys and girls that were injured (p = 0.481) (Table 2). The knee was the most commonly affected body part, accounting for 13 cases (40.6%), followed by the ankle (N = 7, 21.9%). The forearm, wrist and fingers, hip and thigh, and the leg were the least body parts affected, accounting for 1 case each (3.1%) (Table 3). Injuries to the ankle and knee joints occurred more in boys than girls, although, there was no statistically significant difference between them (p = 0.590) (Table 3).

**Table 2:** Types of injuries sustained

**Injury**	**Boys n (%)**	**Girls n (%)**	**Total n (%)**
Contusion	2 (66.7)	1 (33.3)	3 (9.4)
Dislocation	1 (100.0)	- (0.0)	1 (3.1)
Laceration	1 (50.0)	1 (50.0)	2 (6.3)
Sprain	11 (55.0)	9 (45.0)	20 (62.5)
Strain	2 (40.0)	3 (60.0)	5 (15.6)
Cramp	- (0.0)	1 (100.0)	1 (3.1)
Total	17	15	32 (100.0)

*X*^2^ – 4.93, p = 0.424.

**Table 3:** Body parts injured

**Body part injured**	**Boys n (%)**	**Girls n (%)**	**Total n (%)**
Face	2 (100.0)	- (0.0)	2 (6.3)
Abdomen/Trunk	- (0.0)	1 (100.0)	1 (3.1)
Forearm	1 (100.0)	- (0.0)	1 (3.1)
Wrist & Finger	1 (100.0)	- (0.0)	1 (3.1)
Elbow	1 (33.3)	2 (66.7)	3 (9.4)
Hip & Thigh	- (0.0)	1 (100.0)	1 (3.1)
Leg	- (0.0)	1 (100.0)	1 (3.1)
Knee	7 (53.8)	6 (46.2)	13 (40.6)
Ankle	4 (57.1)	3 (42.9)	7 (21.9)
Toes	1 (50.0)	1 (50.0)	2 (6.3)
Total	17	15	32 (100.0)

*X*^2^ – 7.46, p = 0.590.

### Zone of the court where injury occurred

Majority of the injuries occurred in the offensive half (N = 13, 41%). The defensive half and key area of the court accounted for 8 (25%) and 11 (34%) injuries respectively.

### First aid rendered

Cryotherapy (N = 28, 52.8%) was the most frequently administered first aid treatment, followed by strapping/bandaging (N = 15, 28.3%). Bleeding control was the least administered treatment (N = 2, 3.8%) (Table 4). Regarding further care disposition, 75% of the total injured players continued playing after receiving medical attention while 25% could not continue playing despite being treated.

**Table 4:** First aid rendered to the injured players

**Modalities**	**Frequency (n)**	**Percentage (%)**
Cryotherapy	28	52.8
Massage	5	9.4
Stretching	3	5.7
Strapping/Bandaging	15	28.3
Bleeding Control	2	3.8
Total	53	100

## Discussion

The growth and popularity in secondary school (high school) basketball participation in Nigeria creates an urgent need for injury surveillance to identify potential avenues for injury prevention. The present study investigated the incidence and pattern of injuries in a representative sample of adolescent basketball players in Nigeria as a step towards injury prevention.

Injury in previous related studies range from any incident evaluated to incidents that result in at least a day absence and the metric of risk include percentage injured, injuries per match or injuries per 100 or 1,000 hours of exposure or per 1,000 athletes/participants [10-16]. It is important to note that no publication presently exist concerning a consensus statement for basketball injury surveillance. This has resulted in differences in injury definitions and methodologies by different authors. Our definition of injury included both medical attention and time loss injuries. Differences in methodology made it difficult to compare with some studies done in Europe. However, an incidence of 22.7 injuries per 100 participants recorded in this study for each gender falls within the range documented for American public high school which varies considerably from 5.6–36.8 per 100 participants for boys, and 7.8–49 per 100 participants for girls [9]. An incidence of 1.0 injury per match recorded in this study is comparable to 0.7 recorded by Akinbo et al. [6]. This suggests that there is a higher risk of injuries among adolescent amateur basketball players than among the professionals in Nigeria.

This study revealed that jumping/landing and sudden turn, twists or stop were the most common causes of injuries in young basketball players in Nigeria. This is in agreement with other studies on adolescent/youth basketball players [10,14]. However, it contrasts with some other studies [6,16] on professional male and female basketball players in which collision with an obstacle (fellow player or opponent) was documented as the major cause of injuries. This contrast may be due to the difference in the levels of play and skills.

Sprain was predominantly the most common type of injury documented for both genders which was more represented at the knee and ankle joints. This is consistent with other previous epidemiological studies on adolescent and professional basketball players [6,10-14]. Jumping/landing caused majority of the knee and ankle ligament sprains documented in this study. Intervention programs that focus on jumping or balance training in these players could prove to be effective injury prevention strategy [17,18].

The present study revealed that the offensive zone recorded the highest injuries of the three zones followed by the key area and then the defensive half. This agrees with other previous studies [6,9,14]. A high incidence of injuries in this zone is probably because it is a very active area where offensive players try to make shots against their opponents followed by the key area where they struggle to get rebounds in other to make a shot or save the ball from getting into their opponent possession.

Concerning body part affectation, the lower limbs, represented mostly by the knee joint accounted for 75% of all injuries. This is consistent with previous studies on basketball [9-16]. There are still some controversies on the most frequently injured anatomical site in basketball. While some authors [15,16] reported the knee as most commonly injured body part, most other authors [9-14] reported the ankle to be the most commonly injured body site. Repetitive jumping in basketball imposes recurring consistent vertical ground reaction forces of up to four times body weight on the weight-bearing knee joint [19]. The maturing (adolescent) neuromuscular system may be unable to maintain knee stability and around-joint control, leading to forces above the physiological threshold, with inevitable injury to the knee joint structures.

The most frequently used treatment modalities were cryotherapy and bandaging. Cryotherapy in the form of cold compress or cold spray application remains the mainstay first aid treatment for most acute on-field injuries. Over 50% of treatments given during the competition involved Cryotherapy. This corroborates other studies [6,7,20,21]. This is because many of the injuries were minor ligament sprains and muscle strains which did not require any form of special treatment modalities. However, some (25%) of the injuries were time loss injuries. This implies that 1 out of every 4 injuries resulted in a disposition of discontinuity of play during the competition. These time loss injuries were moderate and severe injuries that prevented players from returning to play immediately after a sideline treatment. It is imperative that injury countermeasures that would help prevent such injuries in future competitions are introduced.

The level and intensity of play in high risk sports such as basketball, is one area which may be addressed by sports medicine experts, parents, coaches, sports administrators and injury prevention policy makers. The focus of sports activity by young people should be directed at technique development in order to train their neuromuscular system to control the biomechanical strain imposed by the game of basketball.

## Conclusion

The incidence of match injuries in adolescent amateur basketball players during a national competition in Nigeria was 22.7 injuries per 100 participants; equivalence of 1.0 injury per match. The pattern of injuries was similar in both genders and consistent with what has been previously reported in literature for adolescent basketball. Majority of the injuries were to the lower extremities and knee ligament sprains were the most reported injuries.

Continued surveillance of nationally representative samples of Nigeria’s young basketball players is essential to fully understand the injuries in this population. The increased quantity of data will aid in the development of precise preventive methods and also help in measuring the impact of any interventions. Exercise-based injury prevention programmes aimed at improving core strength and neuromuscular control at the lower limbs may help reduce the incidence of injuries.

## Competing interests

No competing interests.

## Authors’ contributions

OBA was involved with study concept and design, analysis of data, interpretation of data, and preparation of the manuscript. AK was involved with study design, interpretation of data, and drafting of manuscript. BM was involved in study design, acquisition of data, analysis of data and drafting of manuscript. SRA was involved with study concept and design, and interpretation of data. All authors were involved with critical revision of the manuscript for important intellectual content and approved the final version of the manuscript.
